# Health workers' attitudes toward sexual and reproductive health services for unmarried adolescents in Ethiopia

**DOI:** 10.1186/1742-4755-9-19

**Published:** 2012-09-03

**Authors:** Mesfin Tilahun, Bezatu Mengistie, Gudina Egata, Ayalu A Reda

**Affiliations:** 1 School of Graduate studies and College of Health Sciences, Haramaya University, Harar, Ethiopia; 2 Kersa Woreda Health Bureau, Eastern Haraghe Zone, Hararghe, Oromia, Ethiopia; 3 Department of Environmental Health Science, College of Health Sciences, Haramaya University, Harar, Ethiopia; 4 Department of Public Health, College of Health Sciences, Haramaya University, Haramaya, Ethiopia; 5 Population Studies and Training Center, Brown University, Providence, RI, USA; 6 Department of Sociology, Brown University, Providence, RI, USA

## Abstract

**Background:**

Adolescents in developing countries face a range of sexual and reproductive health problems. Lack of health care service for reproductive health or difficulty in accessing them are among them. In this study we aimed to examine health care workers' attitudes toward sexual and reproductive health services to unmarried adolescents in Ethiopia.

**Methods:**

We conducted a descriptive cross-sectional survey among 423 health care service providers working in eastern Ethiopia in 2010. A pre-tested structured questionnaire was used to collect data. Descriptive statistics, chi-square tests and logistic regression were performed to drive proportions and associations.

**Results:**

The majority of health workers had positive attitudes. However, nearly one third (30%) of health care workers had negative attitudes toward providing RH services to unmarried adolescents. Close to half (46.5%) of the respondents had unfavorable responses toward providing family planning to unmarried adolescents. About 13% of health workers agreed to setting up penal rules and regulations against adolescents that practice pre-marital sexual intercourse. The multivariate analysis indicated that being married (OR 2.15; 95% CI 1.44 - 3.06), lower education level (OR 1.45; 95% CI 1.04 - 1.99), being a health extension worker (OR 2.49; 95% CI 1.43 - 4.35), lack of training on reproductive health services (OR 5.27; 95% CI 1.51 - 5.89) to be significantly associated with negative attitudes toward provision of sexual and reproductive services to adolescents.

**Conclusions:**

The majority of the health workers had generally positive attitudes toward sexual and reproductive health to adolescents. However, a minority has displayed negatives attitudes. Such negative attitudes will be barriers to service utilization by adolescents and hampers the efforts to reduce sexually transmitted infections and unwanted pregnancies among unmarried adolescents. We therefore call for a targeted effort toward alleviating negative attitudes toward adolescent-friendly reproductive health service and re-enforcing the positive ones.

## Introduction

According to World Health Organization (WHO) definition adolescent comprises individuals between the age group of 10–19 years [1]. It is the period of transition from childhood to adulthood characterized by significant physiological, psychological and social changes [1,2].

Adolescents suffer from life threatening health risks related to early marriage, unwanted pregnancies, unsafe abortions, sexually transmitted infections (STIs) including HIV/AIDS, female genital mutilation, malnutrition and anemia, infertility, sexual and gender based violence, and other serious reproductive health and social problems. Many adolescents die prematurely. An estimated 70,000 teenage girls die every year during pregnancy and childbirth and more than one million infants born to adolescent girls die before their first birthday [3-6].

An estimated 14 million adolescents give birth globally each year and more than 90% of these live births occur in developing countries. Adolescents in the Sub-Saharan Africa region have low family planning utilization rates and limited knowledge of reproductive health (RH) services. They account for a higher proportion of the region’s new HIV infections, maternal mortality, and unmet need for reproductive health information and services which is linked to social, cultural, economic and gender related factors [4,7].

The literatures shows that adolescents often lack basic RH information, knowledge, experience, and are less comfortable accessing reproductive and sexual health services than adults. This could be attributed to parents, health care workers, and educators who are frequently unwilling or unable to provide age-appropriate RH information to young people [8]. This is often due to their discomfort about the subject or the false belief that providing the information will encourage sexual activity. Adolescents’ embarrassment or discomfort to discuss sensitive topics with their health care provider, less favorable attitudes toward the use of health services and providers, disappointment with how health care providers questions, uncertainty on what providers do with information, and being treated disrespectfully and even denial of the service by their health care providers are often cited as discouraging [4,7,9].

In Ethiopia, youth commonly suffer from reproductive health problems such as sexual coercion, early marriage, female genital cutting, and sexually transmitted infections. According to the 2011 EDHS, 28.6% of the married women were using family planning method. The coverage is only 23.8% among adolescents’ of 15–19 years of age. Unmet need for family planning in Ethiopia in the same year was 25% and it is highest among adolescents of 15–19 years of age. Although the government provides contraception at no cost, these supplies are frequently not readily accessible. Childbearing also begins early, with 45% of total births in the country occurring among adolescent girls and young women [10-12].

Reports indicate that demand for sexual and reproductive health services by adolescents is increasing in developing countries [13-15]. However, there is limited evidence on the provision of the service, its effectiveness, and the role of the different stakeholders involved [13,14]. Integrated services delivered through the healthcare system are identified as one of the most effective ways of delivering RH services [16]. Health professional are responsible to promote and provide the sexual and reproductive health service to adolescents in health facilities. The evidence in many countries has shown that most young people do not routinely seek sexual and reproductive health service. The role of health professionals as a source of information is found to be low [17]. In order to provide the service it is imperative that providers themselves should have positive attitude towards the service. Little is known about health workers attitude towards sexual and reproductive health services for unmarried adolescents in Ethiopia. The study will give insight about health care workers’ attitudes toward adolescent sexual and reproductive health and could be helpful to design appropriate intervention measures to improve adolescent sexual and reproductive health in the country.

## Methods

### Settings and study design

Ethiopian health care institutions are structured according to the World Health Organization’s recommendation for primary health care [18] and consist of community health centers and hospitals with governmental and private ownership. The institutions included in this study provide service to more than 3 million people residing in urban and rural areas [19]. Contraception including primarily, pills, injection, emergency contraception and counseling services are provided for clients. Services like intra-uterine devices, Norplant and tubal ligation are provided at the higher centers like hospitals. There are no specialized family planning workers in Ethiopia. Instead, and as seen practically in our study area, all health care workers are responsible for working on RH services department of the health institutions. Mostly they work in rotations that may range from a month to a year.

We conducted a cross-sectional survey among 423 (15.5%) of the 1704 health workers working in two hospitals and 83 health centers in eastern Hararghe, Ethiopia (Oromia region) using a stratified proportional sampling procedure in which samples were drawn from each health institution in proportion to the number of health workers at the time of the study. The sample size was calculated using the formula for estimation of a single proportion [20], n = z^2^*p(1-p)/r^2^. Where the z value is taken as 1.96; p, proportion of positive attitudes, was assumed to be 50%; and r, the margin of error of estimation, was assumed to be 5% or 0.05. This provided a sample size of 384. To account for non-response 10% was added, providing a sample size of 423. All health care personnel including physicians, nurses and health assistants, working in the institutions and directly involved in day-to-day patient care and services were included in the study. The researchers reached participants through their respective institution and department heads. Data collection took place from August to October, 2010.

### Questionnaire and data collection

Data were collected using a self-administered structured questionnaire provided to respondents at their respective health institutions. It was developed after reviewing qualitative and quantitative research in the area of family planning and adolescent reproductive health. Final items were generated after discussion among the researchers. After consensus, the items were checked for clarity and translated into the local language of Oromiffa. The resulting questionnaire was pretested on a convenience sample of 20 health workers that were not included in the study and corrections were made afterwards. The final questionnaire contained items on basic demographic information such as age and sex; and perception and attitudes toward adolescent sexual and reproductive health. Most of the attitude questions were rated into three responses - agree, disagree, and neutral.

### Statistical analysis

Questionnaires were checked for completeness and consistency and then entered into EPI INFO software version 3.5.1, corrected and cleaned. The data were then transferred to IBM® SPSS® Statistics, version 16 for Windows for analysis. Chi-square tests and simultaneous entry multivariable logistic regression were performed to examine associations. Unadjusted and adjusted (AOR) odds ratios were used as indicators of the strength of association. In the analysis a conservative approach was followed in which disagreement and neutral attitude were merged together. The cut-off level for alpha was set at 0.05.

### Operational definitions

In this study adolescent refers to young persons of both sexes in the age interval of 11 to 19. Furthermore they must not be in a union which has acceptance by the community or is considered a legal marriage. Health workers refers to a health professional working in the study area at the time of data collection and having certification to work in health service institutions in direct care of patients including provision of family planning or related reproductive health services.

### Ethical clearance

The Institutional Research Ethics Review Committee of Haramaya University provided ethical approval. The health workers were provided information about the study and its importance, and confidentiality of the information requested. Written consent was then obtained from participants in a form provided with the study questionnaire.

## Results

Out of the total 423 health workers contacted for interviews, 401 (94.8%) respondents gave responses. Seven questionnaires with incomplete and inconsistent responses were excluded. The analysis was conducted on information collected from the remaining 394 (93.1%) participants.

### Characteristics of respondents

About half of the respondents belonged to the age range of 18–24 years (219, 55.6%) and the majority (301, 76.4%) were females. The sample comprised two hundred thirty six (59.5%) health extension workers, 119 (30.2%) nurses, 21 (5.3%) health assistants among others responsible for delivering reproductive health services (Table 1). About 42% (166) of the health workers were using some form of family planning at the time of the study. Two hundred and eighty nine (73.3%) participants reported to have taken some form of training on sexual and reproductive health services after graduation.

**Table 1:** **Socio-demographic characteristics of the studied subjects, east Hararghe, Ethiopia**^**§**^

**Characteristics of respondents**	**Frequency**	**Percent**
Age (in years)		
18-24	219	55.6
25-35	143	36.3
36 and above	32	8.1
Sex		
Male	93	23.6
Female	301	76.4
Married		
Yes	245	62.3
No	149	37.7
Education		
Certificate	254	64.5
Diploma and above	140	35.5
Religion		
Muslim	214	54.3
Orthodox	145	36.7
Others	35	9.0
Service time		
< 10 years	338	85.8
10-20 years	51	12.9
> 20 years	5	1.25
Residence		
Rural area	283	71.8
Urban area	111	28.2
Health institution		
Health offices	41	10.4
Hospitals	9	2.3
Health centers	87	22.1
Health stations	23	5.8
Health posts	234	59.4

^§^Proportions were calculated from valid responses, excluding missing values.

### Attitudes of the HCWs

The majority of health workers had positive attitudes toward providing sexual and reproductive health services to unmarried adolescents; however, a significant minority had negative attitudes. One hundred twenty one (30.7%) respondents showed unfavorable attitudes toward providing sexual and reproductive health services (RH) for adolescents. Seventy one health workers (19%) disagreed with expanding the services beyond the health facilities where it is convenient to access a large number of adolescents. Fifty (12.7%) disagreed with the capability of health workers to improve the reproductive health needs of adolescents, whereas 190 (48.2%) believed in options other than reproductive health services to solve the problem. One of the options included punishing adolescents that practice premarital sexual intercourse. Almost half disagreed in accepting the importance of the services to prevent unwanted pregnancy. Also 181 (46.5%) gave unfavorable responses when asked to express their preference to provide family planning (FP) services to adolescents. About 13% argued to set up and apply penal rules and regulations against pre-marital sex practicing adolescents, and 18% believed in strict control of the adolescents, especially toward females. Two hundred fourteen (54.1%) said that they would have negative attitudes towards their own daughters or close female relatives if they came across the information that they were using family planning methods. When compared with the same case for males, 40% showed disapproval. Two hundred twenty eight (57.9%) respondents reported that they have never used family planning services themselves; about ninety seven of these (24.6%) were in marital union.

Three hundred thirty two (84.30%) gave positive attitude on the importance of adolescents’ active participation in reducing their reproductive health problems. Eighty (20.3%) and 40 (10.2%) health workers reported neutral and negative attitudes towards awareness creation to adolescents about practicing safe sex, respectively (Table 2).

**Table 2:** **Responses of health care workers concerning sexual and reproductive health services for adolescents, east Hararghe, Ethiopia**^**¥**^

**Items assessing health workers' attitudes**	**Responses**		
	**Positive, n (%)**	**Neutral, n (%)**	**Negative, n (%)**
Intention on SRHS expansion for UAs	328 (83.2)	44 (11.3)	22 (5.5)
Health workers’ importance in reducing ASRH problems	319 (80.9)	50(12.9)	25 (6.3)
SRHS expansion is crucial issue for female UAs	262 (64.0)	123 (31.2)	19 (4.8)
Adolescents’ active participation is important in reducing SRH related problems of the premarital adolescents	332 (84.3)	41 (10.4)	21 (5.3)
Discussion between parents and UAs on SRH is mandatory to reduce and control SRH problems of the UAs	321 (81.4)	38 (9.6)	35 (8.9)
Awareness creation to UAs about skills of practicing safe sex negotiation is one step to reduce UASRH problems	274 (62.7)	80 (20.3)	40 (10.2)
UAs have harder time to get SRHS than married clients	285 (72.3)	85 (21.5)	24 (6.1)
UASRHS is important only for female adolescents b/c they are the only victims of the SRH problems	159 (40.3)	210 (53.3)	25 (6.3)
Sex education is better to be started at pre-adolescence age	148 (37.5)	43 (10.9)	193 (4.9)
ASRH service expansion beyond health facilities such as schools and youth centers where a large number of adolescents can be addressed helps to reduce the problem.	235 (59.6)	84 (21.3)	75 (19.0)
ASRH service expansion is an effective way to prevent unwanted pregnancy and its adverse consequences	329 (83.5)	62 (15.7)	3 (8.0)
Adolescents have a right to use FP as that of all other married clients	198 (50.2)	146 (37.0)	50 (12.7)
Pre-marital unsafe abortion cases should not blamed as guilty or the responsible persons for the problem	271 (68.8)	77 (19.5)	46 (11.7)
The way respondents feel towards their adolescent daughters’ contraceptive usage.	180 (45.7)	182 (46.2)	32 (8.1)
The way respondents feel towards their adolescent sons’ contraceptive usage.	236 (59.9)	91 (23.1)	67 (17.0)
The way respondents expect about their spouse’s perception on their adolescent daughter’s contraceptive method usage.	178 (45.2)	156 (39.6)	60 (15.2)
Respondents’ likely to provide FP and other SRH services for every adolescents in future.	256 (65.0)	93 (23.6)	45 (11.4)

^¥^Proportions were calculated from valid values by excluding missing values. Abbreviations used in the table: SHRS, sexual and reproductive health service; UA, unmarried adolescents; UASRH, unmarried adolescent sexual and reproductive health; SRH, sexual and reproductive health; ASRH, adolescent reproductive health. FP, family planning.

### Predictors of negative attitudes toward adolescent sexual and reproductive health

Both bivariate and multivariable analyses were conducted to examine the predictors of negative attitude toward RH services. The multivariate analysis indicated that being married (OR 2.15; 95% CI 1.44 - 3.06), lower education level (OR 1.45; 95% CI 1.04 - 1.99), being a health extension worker (OR 2.49; 95% CI 1.43 - 4.35), lack of training on RH services (OR 5.27; 95% CI 1.51 - 5.89) and participants that do not use family planning (OR 1.77; 95% CI 1.05 - 2.77) were significantly associated with negative attitudes toward provision of sexual and reproductive health services to adolescents (Table 3).

**Table 3:** Studied health workers’ attitude towards sexual and reproductive health services for adolescents, by their selected characteristics, east Hararghe, Ethiopia, 2010

**Explanatory variable**	**Unadjusted OR (95% CI)**	**Adjusted OR (95% CI)**	**p-value**
Age			
18-24	1.0	1.00	
25-30	0.50 (0.30 - 0.84)^*^	0.89 (0.54 - 1.27)	0.45
31-40	0.80 (0.39 - 1.62)	1.02 (0.72 - 1.43)	0.87
> 40	1.05 (0.30 - 3.71)	0.56 (0.30 - 1.03)	0.07
Sex			
Male	1	1.00	
Female	0.75 (0.46 - 1.23)	0.71 (0.42 - 1.23)	0.23
Married			
No	1	1.00	
Yes	9.15 (4.82 - 17.38)^*^	2.15 (1.44 - 3.06)	0.04*
Education			
Certificate	8.47 (3.57 - 20.11)^*^	1.45 (1.04 - 1.99)	0.04*
Diploma	4.99 (1.94 - 12.84)^*^	2.06 (1.20 - 3.56)	0.01*
Degree	1	1	
Religion			
Muslim	1	1.00	
Christian	0.65 (0.41 - 1.03)	0.86 (0.54 - 1.37)	0.54
Others	0.76 (0.34 - 1.66)	0.84 (0.59 - 1.23)	0.37
Profession			
Health extension workers	2.67 (1.57 - 4.55)^*^	2.49 (1.43 - 4.35)	0.01*
Health assistants	2.20 (0.80 - 6.10)	0.86 (0.61 - 1.23)	0.37
Health Officers	0.88 (0.23 - 3.31)	1.68 (1.04 - 2.67)	0.04*
Nurses	1	1	
Specific training on RH services			
Yes	1.00	1.00	
No	4.17 (2.60 - 6.71)^*^	5.27 (1.51 - 5. 89)	0.01*
Service time in years			
< 10	1.00	1.00	
10-20	2.49 (1.38 - 4.47)^*^	1.07 (1.10- 1.45)	0.08
> 20	3.35 (0.88 - 12.74)	0.77 (0.50 - 1.10)	0.28
Involvement in RH provision			
Yes	1.00	1.00	
No	1.03 (0.72 - 2.36)	1.09 (0.79 - 1.47)	0.67
Family planning utilization status			
Yes	1.00	1.00	
No	2.18 (1.38 - 3.44)^*^	1.77 (1.05 - 2.77)	0.03*

## Discussion

Young people make up an important section of the population of developing countries. All over Africa, young people are increasingly practicing pre-marital sexual intercourse [21]. In some countries like Gabon up to 63% of females and 77% of males aged 15–19 have had premarital sexual intercourse. However, the proportion that used condom in the last sexual intercourse was 19% for females and 37% for males [21]. According to the 2011 Ethiopia Demographic and Health Survey, among never married young persons of 15–24 years, about 12.7% of males and 5.6% of females have had sexual intercourse. Among those with a history of sexual intercourse, half of the young men and one third of the young women reported to have used a condom in their recent sexual activity [10]. As a consequence of this, adolescents are vulnerable to a range of reproductive health problems, which run the gamut from sexually transmitted infections such as HIV/AIDS to unwanted pregnancy and unsafe abortions [22]. However, reports indicate that several barriers are faced by adolescents in accessing health services and that more research is needed is needed in this area [16]. This study aimed to examine health care workers (HCWs) attitudes toward provision of sexual and reproductive health (RH) services to unmarried adolescents.

The findings indicate there is positive attitude by the majority of health care workers in eastern Ethiopia, toward provision of RH services to unmarried adolescents. However a significant minority have reported a negative attitude. About 13% agreed to setting up penal rules and regulations against adolescents that practice pre-marital sexual intercourse. On the other hand, 30.7% of respondents had negative attitudes toward providing RH services to unmarried adolescents. Close to half (46.5%) of the respondents had unfavorable responses toward providing family planning to unmarried adolescents. About one third (30.5%) of the respondents had either negative or neutral attitude toward health education activities to create awareness about safe sex.

A study from China indicated that health care workers are ambivalent about providing sexual and reproductive health services to adolescents [23]. Similar to our findings, about half of the respondents in the Chinese sample responded positively to providing family planning to unmarried adolescents. However, unlike the sample in the current study, they had an overwhelmingly positive (92%) response toward health education, arguing for a more in-depth and explicit information about sexuality. In the same manner, more than 80% of the respondents indicated that they could provide counseling about sex and contraception to those who seek their services. There seems to be an ambivalent attitude among the sample of participants in this study and the Chinese samples. However, in comparison, the participants of this study seem to have a more negative attitude toward RH services and adolescents who use them. This could be because the Chinese study included specialized workforce that works on family planning, where as our study included HCWs with varying training and skills level. On top of this, there may be higher awareness in China on the use of contraceptives through the one child policy. This may imply a need for more training and awareness creation among the health care workers in Ethiopia so as to enhance their existing soft skills toward client interaction and attitudes toward reproductive health services to adolescents.

A review by Tylee and colleagues indicates that adolescents fear scolding by health workers and lack of confidentiality [16]. Health workers may also not have the necessary trainings for effective communication with adolescents. In these situations adolescents were shown to seek help from close friends and siblings and in health institutions far from home. They may also be liable to seek the services of illegal health service providers such as illegal abortions, putting themselves at significant risk [16]. In our study area there are no facilities for school health services nor are there, to our knowledge, efforts at encouraging adolescents to seek sexual and reproductive health services in nearby institutions.

The findings of this study imply that there is a poor level of sexual and reproductive health services for unmarried adolescents in the study area when evaluated in the context of the negative attitudes by health workers. A lot has to be done to address this gap. The services should encompass all aspects of an all rounded reproductive health service including sexual education and easily accessible facilities and supportive health workers. Efforts at tackling the spread of HIV/AIDS should also incorporate reproductive health services. Importantly, there is a need for awareness creation trainings among health workers [13]. According to an intervention aimed at increasing service use by adolescents in Lusaka Zambia by MMari and colleagues, institutions of adolescent friendly services increases service utilization even though not as much as expected [24]. The importance of implementing parental and community mobilization on top of improvements in health care system related factors are also emphasized [24-27].

This study has limitations. Even though HCWs had privacy during administration of the questionnaires, the possibility of social desirability bias could not be excluded. Due to this possibility of under-reporting, we did not examine their practice with regard to providing RH services to adolescents. However, the study has strengths in that it taps into an important research gap in many developing countries. Furthermore we examined a diverse group of health service providers relevant to the setting of a resource poor country.

In conclusion, the majority of the health workers in this study had a positive attitude toward provision of sexual and reproductive health services to unmarried adolescents. However, a minority of them displayed negative attitudes. This is a significant barrier to service utilization by adolescents and hampers the efforts by the government and NGOs to reduce sexually transmitted infections and unwanted pregnancies among unmarried adolescents. We call for a concerted effort toward creating an adolescent-friendly reproductive health service and awareness creation and client handling trainings to health care workers to re-enforce positive attitudes and reduce negative ones. This endeavor should also include adolescents as well as policy makers.

## Competing interests

The authors declare that they have no competing interests.

## Authors’ contributions

MT has taken a lead role in writing the proposal, submission and follow up for ethical review, data collection, data entry, and writing of the preliminary results. MT, BM, and GE participated in the planning of the study. MT and AAR have involved significantly in the analysis and writing of the manuscript. All authors read and approved the final manuscript.

## Authors’ information

MT holds an MPH degree and is a senior public health practitioner at Kersa district health bureau. BM and GE are lecturers and final year PhD candidates in public health at Haramaya University in Ethiopia, their research interests include adolescent, and child and maternal health. AAR worked with Haramaya University in Ethiopia as a lecturer and has involved in surveys, meta-analyses, trials and other large longitudinal studies; he holds degrees in public health and epidemiology and is a PhD candidate in Demography at Brown University, USA; his research interests include HIV/AIDs, adolescent, child and maternal health, and demography.
